# New data on the huntsman spiders (Araneae, Sparassidae) of China

**DOI:** 10.3897/BDJ.13.e153724

**Published:** 2025-04-11

**Authors:** Changhao Hu, He Zhang, Yang Zhong

**Affiliations:** 1 School of Nuclear Technology and Chemistry & Biology, Hubei University of Science and Technology, Xianning, Hubei, China School of Nuclear Technology and Chemistry & Biology, Hubei University of Science and Technology Xianning, Hubei China; 2 Guo Shoujing Innovation College, Xingtai University, Xingtai, Hebei, China Guo Shoujing Innovation College, Xingtai University Xingtai, Hebei China; 3 The Arachnid Resource Centre of Hubei Province & The State Key Laboratory of Biocatalysis and Enzyme Engineering of China & Centre for Behavioural Ecology and Evolution, School of Life Sciences, Hubei University, Wuhan, China The Arachnid Resource Centre of Hubei Province & The State Key Laboratory of Biocatalysis and Enzyme Engineering of China & Centre for Behavioural Ecology and Evolution, School of Life Sciences, Hubei University Wuhan China; 4 Hubei Broad Nature Technology Service Co., Ltd., Wuhan, China Hubei Broad Nature Technology Service Co., Ltd. Wuhan China

**Keywords:** new species, new records, biodiversity, taxonomy, morphology

## Abstract

**Background:**

Sparassidae Bertkau, 1872 is the tenth largest family of Araneae, with 11 genera and 288 species known in China.

**New information:**

In the current paper, four newly-recorded species from China are provided: *Heteropodaacris* Korai & Jäger, 2024, *Oliossericeus* (Kroneberg, 1875), *O.suung* Jäger, 2012 and *Rhitymnaplana* Jäger, 2003, of which *O.sericeus* is re-described, based on specimens collected from Xinjiang Uygur Autonomous Region, China. Additionally, a new *Thelcticopis* species, *T.lini* sp. nov., is described.

## Introduction

The spider family Sparassidae Bertkau, 1872 is well-known for its large size and ferocious habits. As the tenth-largest family of Araneae, Sparassidae contains 1529 species belonging to 98 genera worldwide (World Spider Catalog 2025). Currently, 11 genera and 288 species are recorded in China. Most Chinese genera and species are distributed in southern China, except for *Eusparassus* Simon, 1903 and *Micrommata* Latreille, 1804 from north-western China (Hu and Fu 1985, Song et al. 1999, Jäger 2008, Sun et al. 2011, Quan and Liu 2012, Zhong and Liu 2014, Ding et al. 2019, Zhong et al. 2019, Zhang et al. 2023, Hu and Liu 2024, Lin 2024, Zhang et al. 2024). During the examination of sparassid specimens collected from China, four species newly recorded in China and one new species belonging to the genus *Thelcticopis* Karsch, 1884 were identified. The aims of the current paper are to provide photos of the newly-recorded species and to describe a new *Thelcticopis* species from China.

## Materials and methods

Specimens examined in this study are deposited in the School of Nuclear Technology and Chemistry & Biology, Hubei University of Science and Technology (HUST) in Xianning. Specimens were examined using an Olympus SZX7 stereomicroscope. Photographs were taken with a Leica M205 C stereomicroscope and final multifocal images were produced with Helicon Focus v. 7.7.0 (Khmelik et al. 2021). The male palps were examined and photographed after dissection. The epigynes were dissected from the spiders' body, examined and then treated in a warmed 0.1 mg/ml Protease K solution before study. All morphological measurements were calculated using a Leica M205 C stereomicroscope. Eye diameters were taken at the widest point. Legs and palp measurements are given as total length (femur, patella, tibia, metatarsus [absent in palp], tarsus). Spination follows that given in Davies (1994). The map was created with ArcGis v. 10.8.1 (Esri 2020). The terminologies used in the text follow Jäger (2020) and Sankaran et al. (2024). All measurements were in millimetres (mm).

Abbreviations: AB = anterior bands; ALE = anterior lateral eye; AME = anterior median eye; C = conductor; CD = copulatory duct; CO = copulatory opening; E = embolus; EA = embolic apophysis; FD = fertilisation duct; Fe = femur; FW = first winding; GP = glandular pore; LL = lateral lobe; MS = median septum; Mt = metatarsus; O = basal embolic outgrowth; Pa = patella; PLE = posterior lateral eye; PME = posterior median eye; RTA = retrolateral tibial apophysis; Sp = spermophor; SS = slit sensillum; ST = subtegulum; TA = tegular apophysis; Ti = tibia; I, II, III, IV = legs I–IV.

## Taxon treatments

### Heteropoda acris

Korai & Jäger, 2024

CDE1F1FB-54EA-5542-85B3-2D718C97A88C

Heteropoda acris : Korai and Jäger (2024): 242, figs. 1A–F, 2A–I, 3A–K, 4A–B and D–E (description of male and female).

#### Materials

- **Type status:** Other material. **Occurrence:** recordedBy: Xiaoran Zhu, Zihao Shen & Likun Dong; individualCount: 1; sex: female; lifeStage: adult; **Location:** continent: Asia; country: China; countryCode: CN; stateProvince: Guangxi Zhuang Autonomous Region; county: Fangchenggang City, Shangsi County; verbatimLocality: Shiwandashan National Nature Reserve, Huangpaoshan scenic area; verbatimElevation: 230 m; verbatimLatitude: 21°57’35”N; verbatimLongitude: 108°02’37”E; **Event:** year: 2024; month: 4; day: 30

#### Description

**Figure 1.**
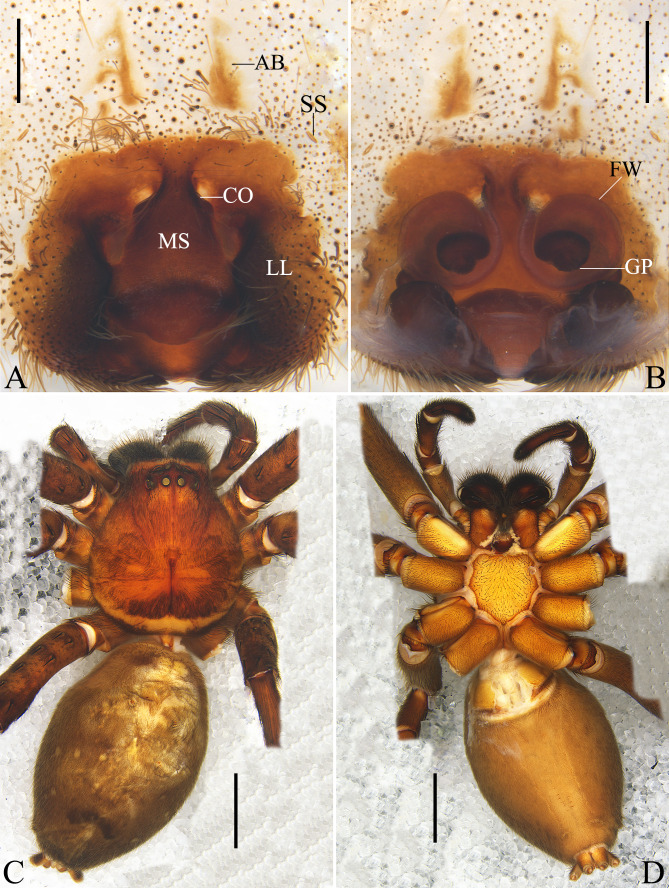
Female of *Heteropodaacris* Korai & Jäger, 2024 from Guangxi Zhuang Autonomous Region, China. **A** epigyne, ventral view; **B** vulva, dorsal view; **C** habitus, dorsal view; **D** habitus, ventral view. Abbreviations: AB anterior bands; CO copulatory opening; FW first winding; GP glandular pore; LL lateral lobe; MS median septum; SS slit sensillum. Scale bars: 0.5 mm (**A, B**); 5 mm (**C, D**).

See Korai and Jäger (2024) (Fig. 1).

#### Diagnosis

See Korai and Jäger (2024).

#### Distribution

China (Guangxi Zhuang Autonomous Region, new record, Fig. 9), Vietnam.

### Olios sericeus

(Kroneberg, 1875)

73ECEBD9-93A4-53F8-ABB1-BF536E99ECFF

Sparassus sericeus : *Kroneberg (1875)*: 28, pl. 3, fig. 19 (description of male and female).
Olios sericeus : *Simon (1880)*: 298; Jäger and Otto (2007): 20, figs. 1–16; Tabrizi et al. (2015): 338, figs. 1–4; Moradmand et al. (2015): 78, figs. 1A–E and 2A–C; Khasayeva and Huseynov (2019): 359, figs. 9–10; Nadolny and Yemets (2024): 11, figs. 1A–G and 2A–S.

#### Materials

- **Type status:** Other material. **Occurrence:** recordedBy: Yaoyu Jiang; individualCount: 2; sex: 1 male 1 female; lifeStage: adult; **Location:** continent: Asia; country: China; countryCode: CN; stateProvince: Xinjiang Uygur Autonomous Region; county: Ili Kazakh Autonomous Prefecture, Yining City; verbatimElevation: 790 m; verbatimLatitude: 43°54’34”N; verbatimLongitude: 81°16’38”E; **Event:** year: 2023; month: 6; day: 25

#### Description

**Male**: Total length 8.5, carapace 3.8 length, 3. 8 width, anterior width of carapace 2.1, opisthosoma 4.7 length, 3.5 width. Eyes AME 0.24, ALE 0.24, PME 0.23, PLE 0.25, AME–AME 0.20, AME–ALE 0.05, PME–PME 0.31, PME–PLE 0.30, AME–PME 0.21, ALE–PLE 0.18, clypeus height of AME 0.10, clypeus height of ALE 0.20. Spination: Palp: 121, 000, 1001; Fe: I–III 323, IV 321; Pa: I–IV 000; Ti: I–III 2124, IV 2024; Mt: I–III 2024, IV 3035. Measurements of palp and legs: Palp 4.8 (1.6, 0.4, 0.9, –, 1.9), I 20.0 (5.6, 1.8, 5.7, 5.2, 1.7), II 22.8 (6.6, 1.9, 6.6, 5.9, 1.8), III 15.2 (4.9, 1.4, 4.1, 3.6, 1.2), IV 17.2 (5.2, 1.5, 4.5, 4.5, 1.5). Leg formula: II-I-IV-III. Chelicerae with two anterior and five posterior teeth.

**Figure 2.**
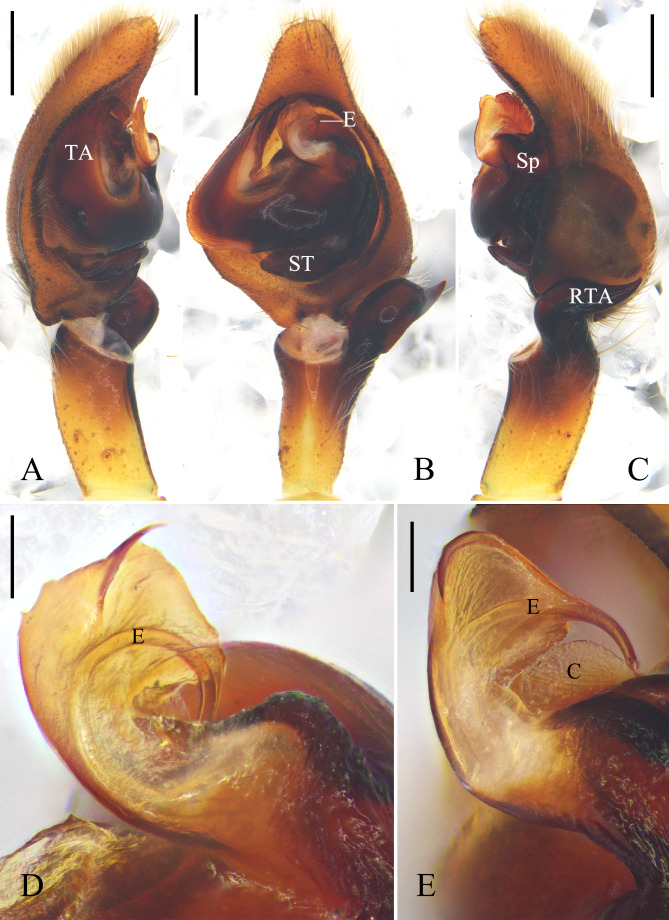
Left male palp of *Oliossericeus* (Kroneberg, 1875) from Xinjiang Uygur Autonomous Region, China. **A** prolateral view; **B** ventral view; **C** retrolateral view; **D** embolus, ventral view; **E** embolus, retrolateral view. Abbreviations: C conductor; E embolus; RTA retrolateral tibial apophysis; Sp spermophor; ST subtegulum; TA tegular apophysis. Scale bars: 0.5 mm (**A–C**); 0.1 mm (**D, E**).

Palp as in diagnosis. Tibia almost three times longer than wide. Retrolateral tibial apophysis (RTA) horn-shaped, extended dorsally, arising from distal tibia. Cymbium almost 1.5 times longer than tibia. Subtegulum (ST) located basal-retrolaterally. Tegular apophysis (TA) massive, with serrated prolateral margin. Conductor (C) semicircular, covered by embolus (E) in ventral view. Embolus (E) curved and small, arising from tegulum at 12 o’clock position, with a small and pointed projection (Fig. 2).

Carapace light yellow, with brown fovea, head region with five brown longitudinal lines. Chelicerae brown, with brown hairs. Sternum light yellow. Endites and labium yellow, with white distal part. Palps and legs orange. Opisthosoma yellow, with brown spots, posterior dorsum with some yellow inverted V-shaped lines, venter with two yellow longitudinal lines. Spinnerets yellow (Fig. 4A and B).

**Female**: Total length 10.3, carapace 4.4 length, 5.0 width, anterior width of carapace 2.9, opisthosoma 5.9 length, 4.0 width. Eyes AME 0.22, ALE 0.24, PME 0.23, PLE 0.23, AME–AME 0.31, AME–ALE 0.20, PME–PME 0.51, PME–PLE 0.39, AME–PME 0.39, ALE–PLE 0.30, clypeus height of AME 0.11, clypeus height of ALE 0.15. Spination: Palp: 131, 001, 1011, 1012; Fe: I 223, II–III 323, IV 321; Pa: I–IV 000; Ti: I–III 2024, IV 2004; Mt: I–III 2024, IV 3035. Measurements of palp and legs: Palp 5.6 (1.7, 0.6, 1.2, –, 2.1), I 19.0 (5.5, 2.1, 5.0, 4.7, 1.7), II 21.2 (6.2, 2.1, 5.8, 5.3, 1.8), III 15.2 (4.7, 1.7, 3.7, 3.7, 1.4), IV 16.7 (5.3, 1.7, 4.1, 4.2, 1.4). Leg formula: II-I-IV-III. Chelicerae with two to three anterior and six to seven posterior teeth.

**Figure 3.**
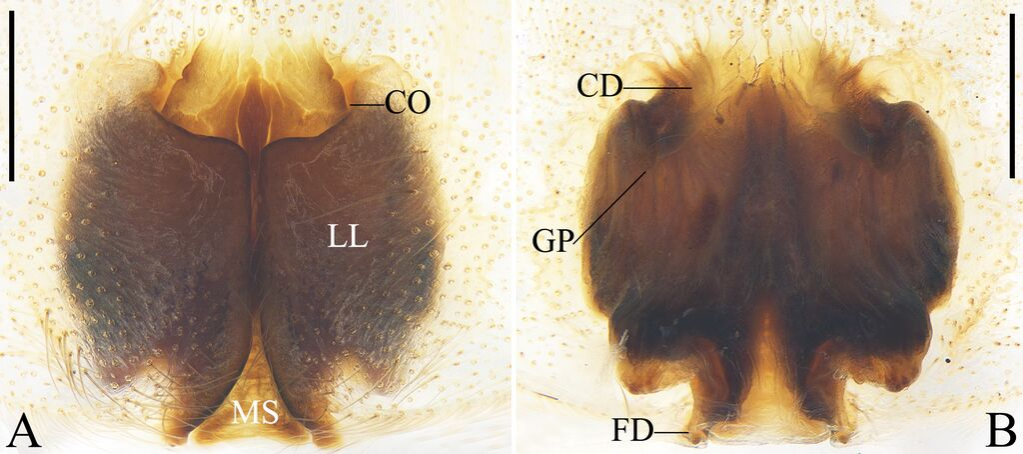
Epigyne of *Oliossericeus* (Kroneberg, 1875) from Xinjiang Uygur Autonomous Region, China. **A** ventral view; **B** dorsal view. Abbreviations: CD copulatory duct; CO copulatory opening; FD fertilisation duct; GP glandular pore; LL lateral lobe; MS median septum. Scale bars: 0.5 mm.

Epigyne as in diagnosis. Epigynal field almost as long as wide. Lateral lobes (LL) almost two thirds length of epigynal field, with wide U-shaped anterior margins and median slit diverging triangularly posterior. Internal duct system with glandular pores (GP) close to copulatory openings (CO). Fertilisation ducts (FD) located posteriorly (Fig. 3).

**Figure 4.**
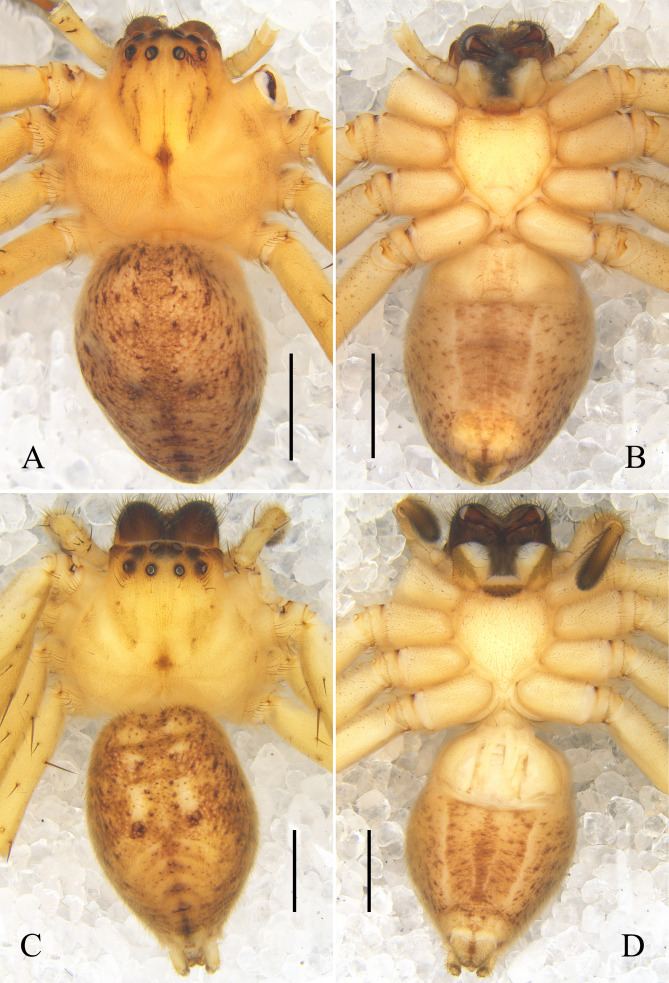
Habitus of *Oliossericeus* (Kroneberg, 1875) from Xinjiang Uygur Autonomous Region, China. **A** male, dorsal view; **B** male, ventral view; **C** female, dorsal view; **D** female, ventral view. Scale bars: 2 mm.

Colouration as in male, generally lighter (Fig. 4C and D).

#### Diagnosis

Males of *Oliossericeus* are similar to those of *O.japonicus* Jäger & Ono, 2000 (cf. Fig. 2 and figs. 10–13 in Jäger and Ono (2000)) and *O.mahabangkawitus* Barrion & Litsinger, 1995 (cf. Fig. 2 and fig. 166b in Barrion and Barrion (1995) and fig. 16 in Jäger and Ono (2000)) in having horn-shaped retrolateral tibial apophysis (RTA), basal-retrolaterally located subtegulum (ST), embolus (E) arising from tegulum at 12 o’clock position, but can be distinguished by: 1. Retrolateral tibial apophysis (RTA) extended dorsally in retrolateral view (vs. extended ventrally in *O.japonicus* and *O.mahabangkawitus*); 2. Tegular apophysis (TA) massive, almost three times wider than embolus (E) (vs. almost as wide as embolus (E) in *O.japonicus* and *O.mahabangkawitus*); and 3. Embolus (E) with a small and pointed projection (vs. absent in *O.japonicus* and *O.mahabangkawitus*). Females of *O.sericeus* are similar to those of *O.rossettii* (Leardi, 1901) (cf. Fig. 3 and figs. 153–155 in Jäger (2020)) in having touching lateral lobes (LL), but can be distinguished by: 1. Anterior margins of lateral lobes (LL) wide U-shaped (vs. V-shaped in *O.rossettii*); and 2. Median slit diverging triangularly posteriorly (vs. not diverging in *O.rossettii*).

#### Distribution

China (Xinjiang Uygur Autonomous Region, new record, Fig. 9), Afghanistan, Azerbaijan, Georgia, Iran, Kazakhstan, Kyrgyzstan, Tajikistan, Turkmenistan, Ukraine, Uzbekistan.

#### Notes

*Oliossericeus* belongs to the *Oliosrossettii*-group, which is characterised by a small U-shaped embolus (E) arising centrally to sub-centrally from the tegulum in males and strongly sclerotised epigyne with a hardly traceable internal duct system in females (Jäger 2020). This species was originally described by Kroneberg 150 years ago based on two male and seven female specimens from Uzbekistan and Kyrgyzstan (Kroneberg 1875); since then, few additional descriptions of this species have been provided (Jäger and Otto 2007, Tabrizi et al. 2015, Moradmand et al. 2015, Nadolny and Yemets 2024). Therefore, we re-describe *O.sericeus*, based on specimens collected from Xinjiang Uygur Autonomous Region, China.

Nadolny and Yemets (2024) noted that specimens of *O.sericeus* from Crimea and Asia show differences in the details of their copulatory organs. Specimens from Crimea have a longer cymbium and the vulva is three times wider than epigynal collar. In contrast, specimens from Asia have a shorter cymbium and the vulva is twice as wide as the epigynal collar. Our specimens from Xinjiang Uygur Autonomous Region, China, match the characteristics of the Asian specimens provided by Nadolny and Yemets (2024).

### Olios suung

Jäger, 2012

B587DD68-C342-56A2-A2C1-E2661B43E1A2

Olios suung : Jäger (2012): 64, figs. 9–12 and 24–26 (description of male).

#### Materials

- **Type status:** Other material. **Occurrence:** recordedBy: Chaotai Wei; individualCount: 1; sex: male; lifeStage: adult; **Location:** continent: Asia; country: China; countryCode: CN; stateProvince: Yunnan Province; county: Xishuangbanna Dai Autonomous Prefecture, Jinghong City; verbatimLocality: rubber forest on the edge of urban area; verbatimElevation: 1318 m; verbatimLatitude: 22°01’18”N; verbatimLongitude: 100°58’21”E; **Event:** year: 2016; month: 3; day: 6

#### Description

**Figure 5.**
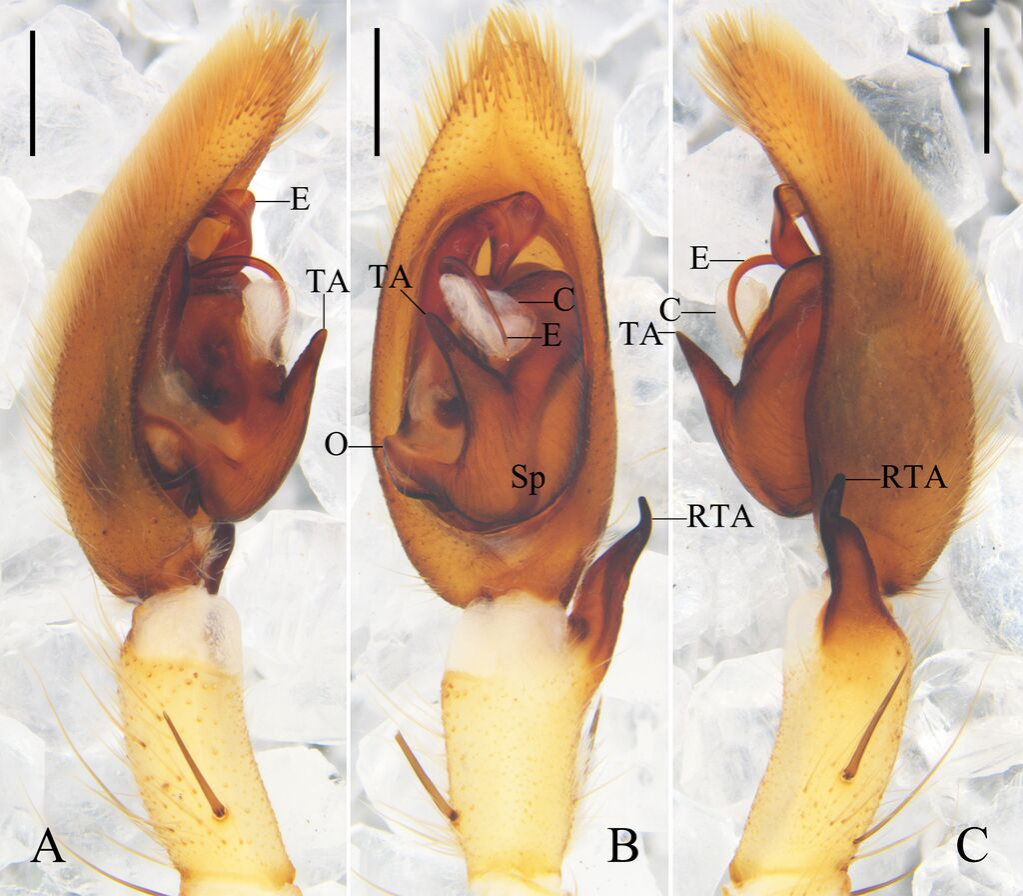
Left male palp of *Oliossuung* Jäger, 2012 from Yunnan Province, China. **A** prolateral view; **B** ventral view; **C** retrolateral view. Abbreviations: C conductor; E embolus; O basal embolic outgrowth; RTA retrolateral tibial apophysis; Sp spermophor; TA tegular apophysis. Scale bars: 0.5 mm.

See Jäger (2012) (Figs 5, 8A and B).

#### Diagnosis

See Jäger (2012).

#### Distribution

China (Yunnan Province, new record, Fig. 9), Laos.

### Rhitymna plana

Jäger, 2003

A246324C-E485-54DF-B95A-9B9041A04D9D

Rhitymna plana : Jäger (2003): 119, figs. 75–82 (description of female); Jäger (2007): 56, figs. 96–97; Jäger (2019): 448, figs. 12–15 and 20–25 (description of male).

#### Materials

- **Type status:** Other material. **Occurrence:** recordedBy: Shengming Liang; individualCount: 1; sex: male; lifeStage: adult; **Location:** continent: Asia; country: China; countryCode: CN; stateProvince: Guangxi Zhuang Autonomous Region; county: Fangchenggang City, Shangsi County; verbatimLocality: Guangxi Shiwandashan National Forest Park; verbatimElevation: 590 m; verbatimLatitude: 21°53’28”N; verbatimLongitude: 107°53’50”E; **Event:** year: 2017; month: 6; day: 7

#### Description

**Figure 6.**
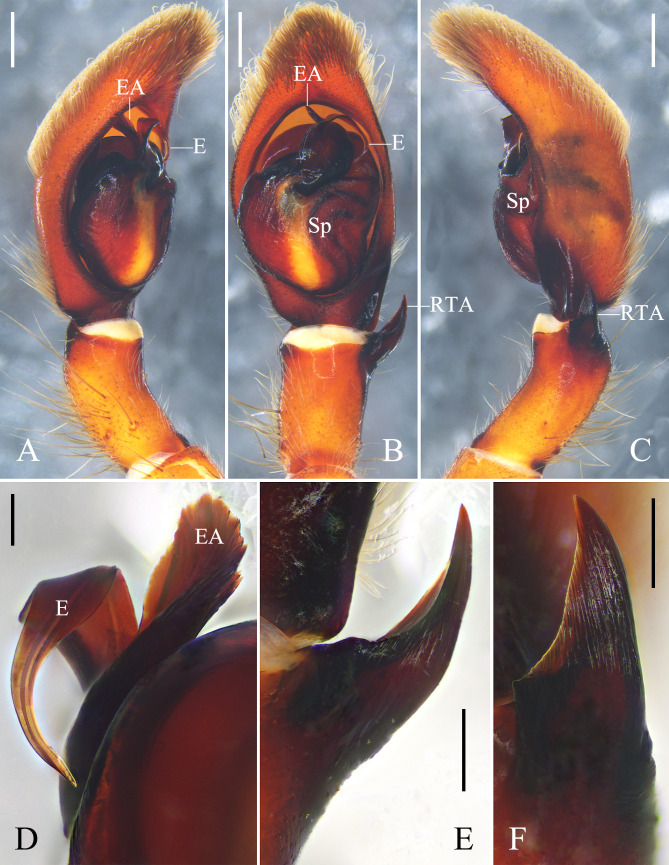
Left male palp of *Rhitymnaplana* Jäger, 2003 from Guangxi Zhuang Autonomous Region, China. **A** prolateral view; **B** ventral view; **C** retrolateral view; **D** embolus and embolic apophysis, retrolateral view; **E** retrolateral tibial apophysis, ventral view; **F** retrolateral tibial apophysis, retrolateral view. Abbreviations: E embolus; EA embolic apophysis; RTA retrolateral tibial apophysis; Sp spermophor. Scale bars: 0.5 mm (**A–C**); 0.1 mm (**D**); 0.2 mm (**E, F**).

See Jäger (2019) (Figs 6, 8C and D).

#### Diagnosis

See Jäger (2019).

#### Distribution

China (Guangxi Zhuang Autonomous Region, new record, Fig. 9), Cambodia, Laos, Vietnam.

### Thelcticopis lini sp. nov.

30CC00D1-8E37-5A79-9ABA-D9C0C75257A3

D2B06A65-9B44-4571-AB53-BEB3BDEAEE6A

#### Materials

- **Type status:** Holotype. **Occurrence:** catalogNumber: HNJFL-18-09; recordedBy: Yejie Lin, Jiaxiang Wu & Rixin Jiang; individualCount: 1; sex: male; lifeStage: adult; **Location:** continent: Asia; country: China; countryCode: CN; stateProvince: Hainan Province; county: Ledong Li Autonomous County; verbatimLocality: Hainan Jianfengling National Forest Park, Mingfenggu; verbatimElevation: 989 m; verbatimLatitude: 18°44’30”N; verbatimLongitude: 108°50’30”E; **Event:** year: 2018; month: 4; day: 20

#### Description

**Male**: Total length 13.9, carapace 7.1 length, 6.2 width, anterior width of carapace 3.4, opisthosoma 7.0 length, 4.6 width. Eyes AME 0.37, ALE 0.25, PME 0.22, PLE 0.26, AME–AME 0.28, AME–ALE 0.28, PME–PME 0.62, PME–PLE 0.69, AME–PME 0.24, ALE–PLE 0.24, clypeus height of AME 0.23, clypeus height of ALE 0.22. Spination: Palp: 131, 101, 3060; Fe: I–II 323, III–IV 321; Pa: I–IV 000; Ti: I–II 212(10), III–IV 2126; Mt: I–II 2022, III 3032, IV 3034. Measurements of palp and legs: Palp 7.2 (2.3, 1.0, 1.3, –, 2.6), I 23,7 (6.2, 2.6, 7.4, 5.8, 1.7), II 23.3 (6.9, 2.7, 6.7, 5.4, 1.6), III 18.4 (5.6, 2.3, 4.7, 4.2, 1.6), IV 23.1 (6.9, 2.2, 5.8, 6.3, 1.9). Leg formula: I-II-IV-III. Chelicerae with three anterior and five to six posterior teeth.

**Figure 7.**
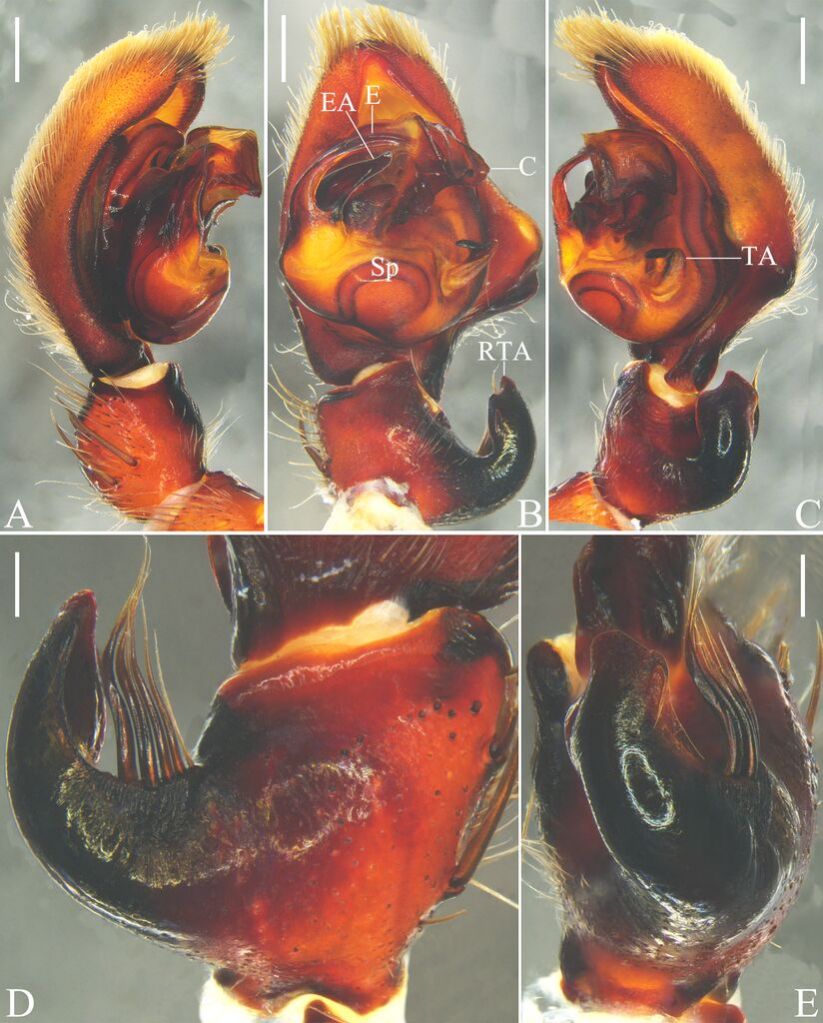
Left male palp of *Thelcticopislini* sp. nov. from Hainan Province, China. **A** prolateral view; **B** ventral view; **C** retrolateral view; **D** tibia, dorsal view; **E** tibia, retrolateral view. Abbreviations: C conductor; E embolus; EA embolic apophysis; RTA retrolateral tibial apophysis; Sp spermophor; TA tegular apophysis. Scale bars: 0.5 mm (**A–C**); 0.2 mm (**D, E**).

Palp as in diagnosis. Tibia almost as long as wide. Retrolateral tibial apophysis (RTA) strongly curved, with irregular-shaped tip; bunch of six to seven setae arising from basal part and one strong seta arising from distal part and elongate ventrally (intersects the margin of retrolateral tibial apophysis (RTA), see Fig. 7E). Cymbium almost twice longer than tibia. Spermophor (Sp) oval in ventral view. Tegular apophysis (TA) straight in retrolateral view, originating from a membrane. Distal conductor (C) rectangular in lateral view. Embolus (E) curved, arising from tegulum at 10 o’clock position, with a lamellar embolic apophysis (EA) on basal embolus (E) (Fig. 7).

**Figure 8.**
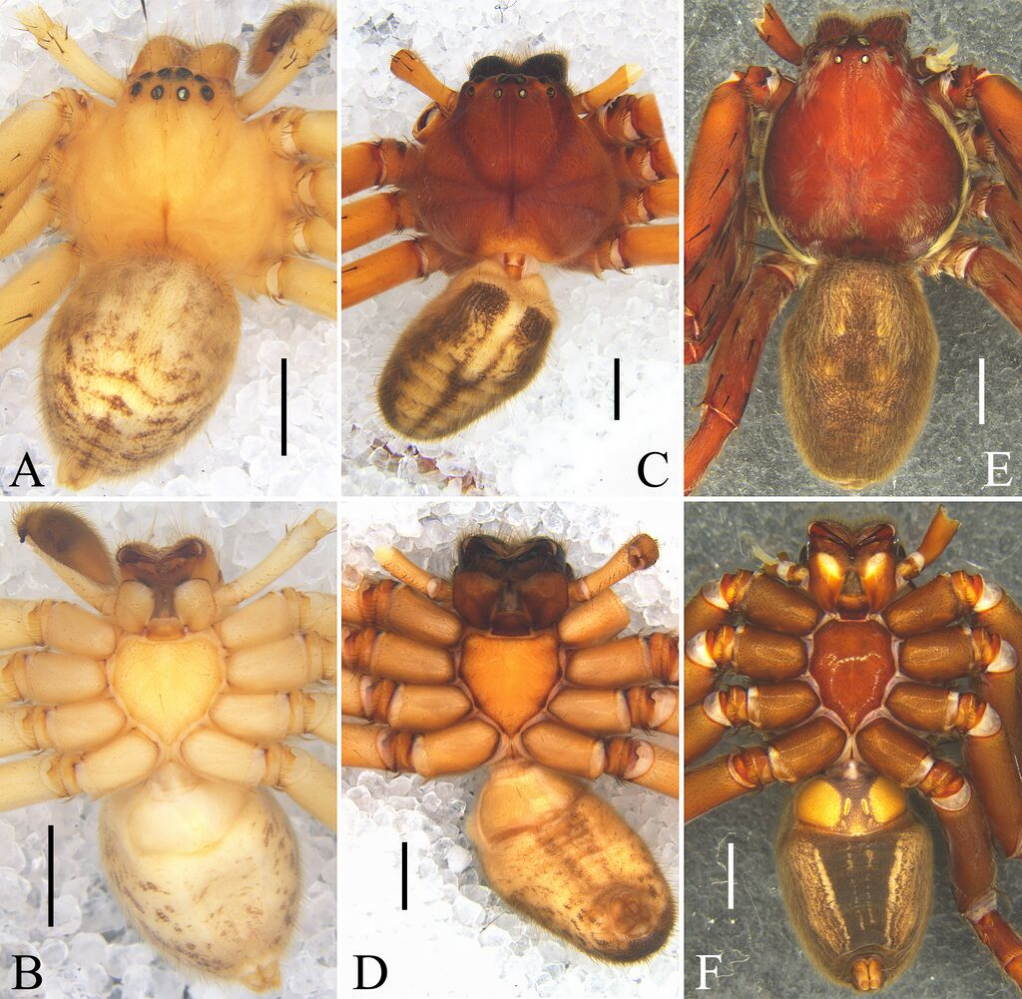
Male habitus. **A, B** *Oliossuung* Jäger, 2012 from Yunnan Province, China; **C, D** *Rhitymnaplana* Jäger, 2003 from Guangxi Zhuang Autonomous Region, China; **E, F** *Thelcticopislini* sp. nov. from Hainan Province, China. **A, C, E** dorsal view; **B, D, F** ventral view. Scale bars: 2 mm.

Carapace reddish-brown, with golden hairs. Chelicerae brown, with brown hairs. Sternum reddish-brown, with brown margin. Endites and labium brown with yellow distal part. Legs brown to yellowish-brown, with golden hairs. Opisthosoma brown, dorsum with dark yellow markings, venter with some yellow longitudinal lines. Spinnerets orange (Fig. 8E and F).

**Female**: unknown.

#### Diagnosis

Males of *Thelcticopislini* sp. nov. are similar to those of *T.severa* (L. Koch, 1875) (cf. Fig. 7 and figs. 2A‒D in Zhu et al. (2020)) and *T.bicornuta* Pocock, 1901 (cf. Fig. 7 and figs. 4E, F, 5A and B in Sankaran et al. (2024)) in having a bunch of strong setae arising from basal retrolateral tibial apophysis (RTA), one strong seta arising from distal retrolateral tibial apophysis (RTA), the tegular apophysis (TA) originating from a membrane and the curved embolus (E) with a lamellar embolic apophysis (EA), but can be distinguished by: 1. The tip of retrolateral tibial apophysis (RTA) irregular-shaped, with three peaks (vs. rounded in *T.severa* and *T.bicornuta*); 2. Tegular apophysis (TA) straight in retrolateral view (vs. hook-shaped in *T.severa* and *T.bicornuta*); and 3. Embolic apophysis (EA) almost four fifths of the length of embolus (E) in ventral view (vs. almost two thirds in *T.severa* and *T.bicornuta*).

#### Etymology

This new species is named after Mr. Yejie Lin (Imperial College London, United Kingdom), who made significant contribution to the taxonomy of spiders.

#### Distribution

**Figure 9.**
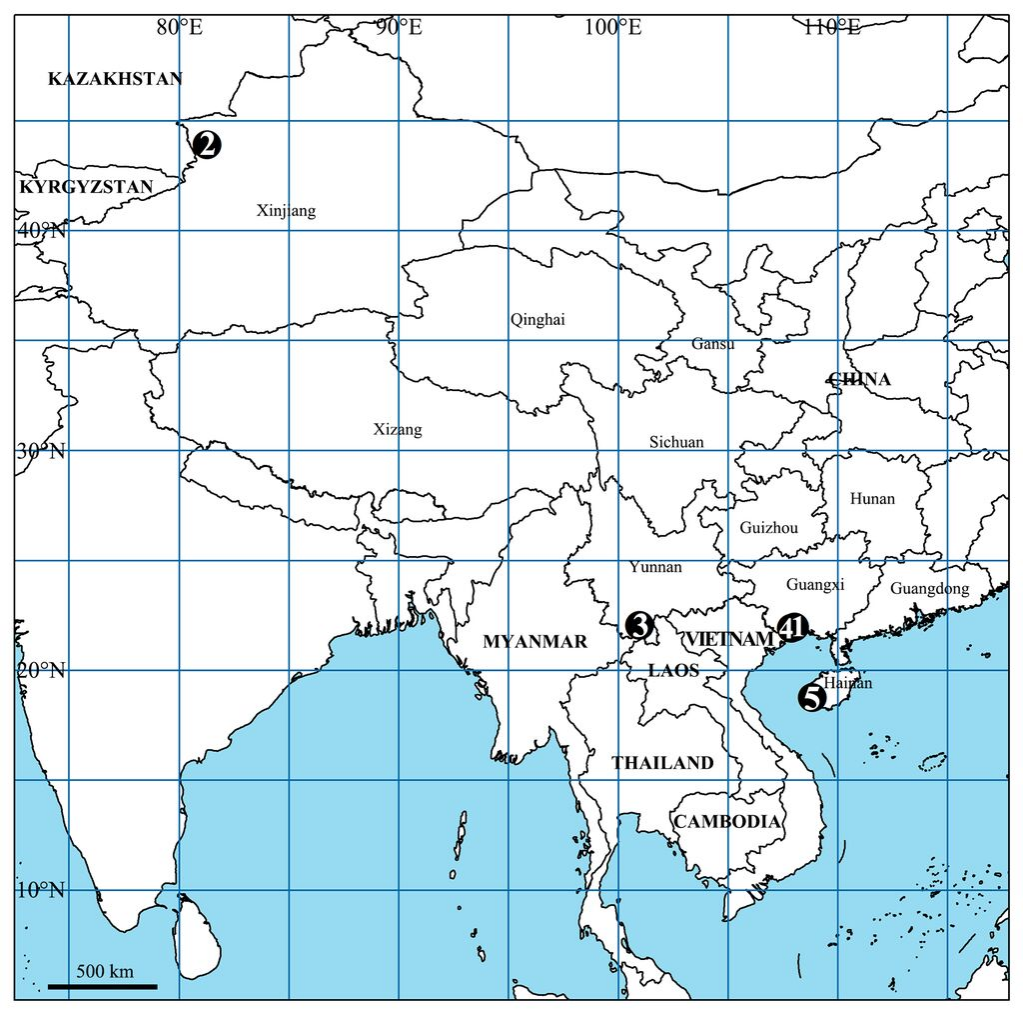
Collection locality of sparassid specimens in the current paper from China. **1** *Heteropodaacris* Korai & Jäger, 2024; **2** *Oliossericeus* (Kroneberg, 1875); **3** *O.suung* Jäger, 2012; **4** *Rhitymnaplana* Jäger, 2003; **5** *Thelcticopislini* sp. nov.

Known only from the type locality (Fig. 9).

## Supplementary Material

XML Treatment for Heteropoda acris

XML Treatment for Olios sericeus

XML Treatment for Olios suung

XML Treatment for Rhitymna plana

XML Treatment for Thelcticopis lini
